# Photocatalytic Hydrogen Production Using TiO_2_‐based Catalysts: A Review

**DOI:** 10.1002/gch2.202400134

**Published:** 2024-10-02

**Authors:** Fahima Bhom, Yusuf Makarfi Isa

**Affiliations:** ^1^ School of Chemical and Metallurgical engineering University of the Witwatersrand Johannesburg 2050 South Africa

**Keywords:** hydrogen production, nanostructures, photocatalytic water splitting, photoreactors, TiO_2_

## Abstract

Photocatalytic water splitting is an environmentally friendly hydrogen production method that uses abundant renewable resources such as water and sunlight. While Titanium dioxide (TiO_2_) photocatalyst exhibits excellent properties, its high band gap limits absorption to ultraviolet (UV) irradiation, resulting in low photo conversion efficiency. This review explores various modification techniques aimed at enhancing the efficiency of TiO_2_ under visible light irradiation. Factors influencing the photocatalytic water splitting reaction, such as catalyst structure, morphology, band gap, sacrificial reagents, light intensity, temperature, and potential of Hydrogen (pH) are examined. This review also summarizes different catalyst synthesis methods, and types of photocatalytic reactors, and provides insights into quantum yield. Finally, the review addresses the challenges and future outlook of photocatalytic water splitting.

## Graphical abstract

Solar and water are critical in addressing the global challenge of energy. Photocatalytic production of hydrogen is a step toward a truly reliable renewable energy source. The paper dwells into the roles of TiO_2_ as a catalyst in the production of hydrogen. Challenges and prospects facing this promising process are also discussed.

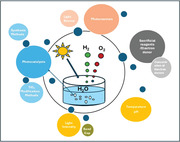

## 1 Introduction

Energy is very essential for a country's socio‐economic development and the well‐being of its people.^[^ ^1^ ^]^ ≈85% of the world's power is produced from fossil fuels.^[^ ^2^ ^]^ The use of non‐renewable energy sources has caused great environmental degradation as burning of fossil fuels releases carbon dioxide (CO_2_), leading to climate change.^[^ ^3^ ^]^ The energy crisis has been caused by the diminishing fossil fuels, a consequence of both population growth and the required industrialization.^[^ ^4^ ^]^ Climate change and energy crises are the two biggest global challenges facing humanity today. To address these challenges and achieve substantial reduction in CO_2_ emissions by 2050, the International Governmental Panel on Climate Change (IPCC) recommended limiting the rise in temperature to no more than 1.5 °C.^[^ ^5^ ^]^ Over the last few years, there has been a lot of effort in finding renewable or alternative energy sources that are clean, sustainable, reduce the energy crises and dependence on fossil fuels.^[^ ^6^ ^]^ Nevertheless, one of the challenges with renewable energy sources is the unpredictable availability of energy because of variability in location and time which may result in the energy not being available at the time of demand; therefore, an energy storage medium is required.^[^ ^3^ ^]^

Hydrogen (H_2_) is considered an optimal energy storage medium due to its cleanliness, abundance, it can be derived from both water and biomass, has high calorific value, and can be stored either as a gas (H_2_) or liquid hydrogen.^[^ ^3^, ^7^ ^]^ However, hydrogen production is associated with high energy consumption, and storage and transportation of hydrogen are costly because of its extremely low volumetric energy density.^[^ ^8^ ^]^ Therefore, researchers are investigating various aspects of hydrogen production, storage, safety, and its role in the energy transition.^[^ ^9^ ^]^ Currently, hydrogen is mainly produced by industrial methods such as steam methane reforming, natural gas decomposition, partial oxidation, and coal gasification.^[^ ^10^ ^]^ However, these methods are not favorable due to their reliance on non‐renewable energy sources.^[^ ^11^ ^]^ Hydrogen production (H_2_) by photocatalytic water splitting (PWS) using renewable resources such as sunlight and water is considered to be a highly promising technology due to its environmentally friendly nature and zero global warming potential.^[^ ^12^ ^]^ Over the past years, numerous semiconductor photocatalysts have been researched and developed for PWS. ^12^ However, PWS is currently in the initial stages of development and requires research advancements in material science and reaction efficiency to become a significant contributor to future sustainable hydrogen production.^[^ ^13^ ^]^

This review aims to offer an overview of the PWS mechanism and explore research conducted on various aspects including types of photocatalysts, methods of modifying Titanium dioxide (TiO_2_) photocatalyst, factors affecting photocatalytic activity, synthesis methods, photoreactor design, quantum yield and challenges as well as future perspectives of PWS.

## 2 Photocatalytic Water Splitting

Photocatalytic water splitting involves dissociation of water into stoichiometric hydrogen (H_2_) and oxygen (O_2_) by harnessing solar energy and converting it into chemical energy with the use of a photocatalyst.^[^ ^14^ ^]^ Photocatalytic water splitting allows the production of hydrogen using the two most abundant renewable sources such as water and sunlight.^[^ ^1^ ^]^ Photocatalysts are semiconductor materials that absorb photons and facilitate the reaction.^[^ ^15^ ^]^ Water splitting is an endothermic process and is characterized by a significant positive change in the Gibbs free energy ($\Delta \;G^{\circ }=\;+\frac{237kJ}{mol},\;2.46\;eV\;per\;molecule)$.^[^ ^16^ ^]^

### 2.1 Photocatalytic Water Splitting Mechanism

**Figure 1:**
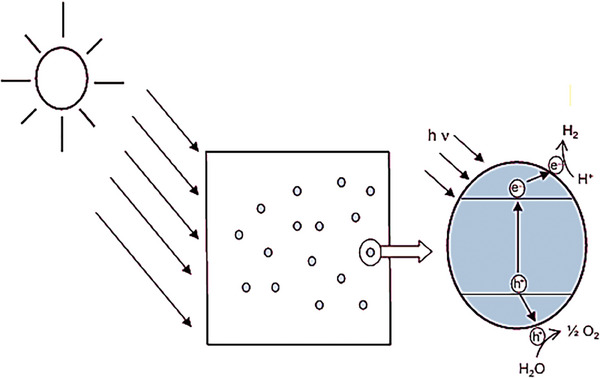
Photocatalytic water splitting mechanism. Reproduced with permission.^[^ ^16^ ^]^ Copyright 2013, Elsevier.

The photocatalytic water‐splitting process involves the following steps: light absorption, charge generation and separation, and redox reactions at the catalyst's surface.^[^ ^17^ ^]^ First, the photocatalyst absorbs light with energy greater than its band gap (*E_g_*) energy.^[^ ^18^ ^]^ The band gap of a photocatalyst refers to the difference in energy levels between its unoccupied conduction band (CB) and electron occupied valence band (VB).^[^ ^14^ ^]^ Upon irradiation, the electrons (e^−^) from the valence band transition to a conduction band forming holes (h^+^) in the VB.^[^ ^16^ ^]^ Eidsvåg et al. reported that based on the $\Delta G^{\circ }$, for a photocatalytic water splitting process a photocatalyst with a bandgap greater than 1.23 eV is essential.^[^ ^6^ ^]^ In the second step, the electron, and holes created move toward the catalyst's surface and react with water in an oxidation and reduction reaction.^[^ ^16^ ^]^ The electron reduces H^+^, while the hole oxidizes H_2_O to produce hydrogen and oxygen, respectively.^[^ ^3^ ^]^ The photocatalytic water splitting mechanism is described by Equations (1)–(4) and the process is illustrated in **Figure** **1**.^[^ ^17^ ^]^

(1) $$\mathrm{Catalyst}\to \mathrm{Catalyst}\left(e^{-}+h^{+}\right)$$

(2) $$\mathrm{Catalyst}\left(e^{-}+h^{+}\right)\to \mathrm{Catalyst}$$

(3) $$H_{2}O+h^{+}\to 1/2O_{2}+2h^{+}$$

(4) $$2e^{-}+2h^{+}\to H_{2}$$

The primary challenge in photocatalytic water splitting is the recombination of electron‐hole pairs (charge) (e^−^ / h^+^), as shown by Equation (2).^[^ ^17^ ^]^ The charge recombination results in no net chemical reaction and hydrogen cannot be produced.^[^ ^3^ ^]^ Eidsvåg et al. reported that the effectiveness of the photocatalyst is maximized in an ideal situation such as in the absence of electron‐hole recombination.^[^ ^6^ ^]^

### 2.2 Types of Photocatalytic Water Splitting Reactions

Photocatalytic water splitting is classified into two types of reactions: 1) Photochemical cell reactions and 2) Photo‐electrochemical cell reactions.

#### 2.2.1 Photochemical‐Cell Reactions

**Figure 2:**
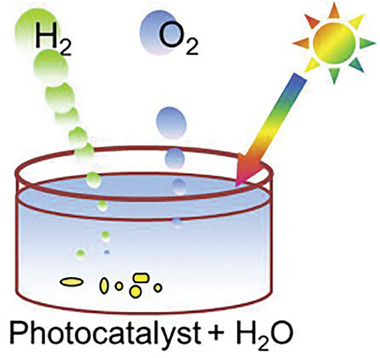
Schematic of a photochemical cell. Reproduced with permission.^[^ ^20^ ^]^ Copyright 2022, Elsevier.

In photochemical cells, water splitting occurs by the direct use of solar energy.^[^ ^19^ ^]^ The powdered photocatalyst is present as dispersed particles in an aqueous solution, this allows particles to function as micro photoelectrode that performs redox reactions to produce hydrogen and oxygen (**Figure** **2**).^[^ ^16^ ^]^ According to Liao et al., the benefit of photochemical cell is that the suspended photocatalyst particles maximizes the available surface area for efficient photocatalytic reactions.^[^ ^3^ ^]^

#### 2.2.2 Photoelectrochemical‐Cell Reactions

**Figure 3:**
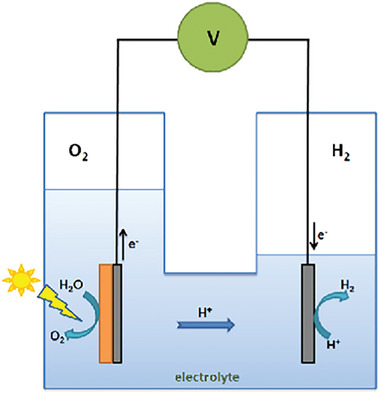
Schematic of a Photoelectrochemical cell. Reproduced with permission.^[^ ^21^ ^]^ Copyright 2015, Elsevier.

Fujishima and Honda in 1972 pioneered the production of hydrogen via photocatalytic water splitting in a photoelectrochemical cell.^[^ ^7^ ^]^ In Photoelectrochemical cells (PEC), as opposed to photochemical cells, a thin layer of photocatalyst is applied on either one or two electrodes to form photoelectrodes which are submerged in an aqueous electrolyte.^[^ ^3^ ^]^ The photoelectrode is exposed to light to conduct the water‐splitting reactions.^[^ ^16^ ^]^ This process requires an additional source of power to electrolyze water before generating hydrogen.^[^ ^14^ ^]^ As shown in **Figure** **3**, the generated electrons are transferred from the photo‐anode to a cathode, leading to the generation of hydrogen, this chemical reaction results in an electric current to flow through the external circuit.^[^ ^21^ ^]^ Pareek et al. reported that this technology is still not commercially viable and requires research on suitable semiconductors and the design of the reactors.^[^ ^1^ ^]^ Jiang et al. studied photoelectrochemical cell reactions and identified challenges in controlling various parameters, such as light absorbance by the photocatalyst, determining instantaneous substrate concentration, and maintaining pH of the reaction solution during the process.^[^ ^22^ ^]^ Ahmad et al. reported that these factors significantly affect the kinetic behavior which complicates the interpretation of the experimental findings.^[^ ^21^ ^]^

Water splitting in a photochemical cell is a simple process as it does not require expensive equipment and it is easy to use.^[^ ^3^ ^]^ The disadvantage of a photochemical cell includes easy recombination of oxygen and hydrogen, whereas in photoelectrochemical cells, efficient separation of oxygen and hydrogen can be achieved enhancing the photocatalytic activity.^[^ ^16^ ^]^ Additionally, Saddique et al. reported that photochemical water splitting is a more viable option than photoelectrochemical water splitting due to its performance, cost‐effectiveness, and environmental friendliness.^[^ ^14^ ^]^ The schematic diagrams of both cells are given in Figures 2 and 3.

## 3 Photocatalysis

Photocatalysis refers to the chemical reaction that occurs as a result of photoirradiation in the presence of a photocatalyst.^[^ ^3^ ^]^ The two types of photocatalysis processes are 1) homogeneous photocatalysis, in which reactant and photocatalyst are in the same phase, and 2) heterogeneous photocatalysis, in which the reactant and photocatalyst are in different phases.^[^ ^15^ ^]^ Sudhir et al. reported that materials with semiconductor properties are commonly utilized as photocatalysts for light redox reactions driven by light.^[^ ^23^ ^]^ The working principle of a photocatalyst has been discussed in Section 2.1 of this work. Applications of photocatalysts include water purification, photocatalytic hydrogen production, controlling odor, deactivating cancer cells, and eliminating bacteria.^[^ ^24^ ^]^

### 3.1 Types of Photocatalysts

For effective photocatalytic water splitting, it is crucial to have a photocatalyst or semiconducting material with suitable properties such as conductivity, redox potential, band gap, non‐toxicity, light absorption, cost‐effectiveness, scalability, and fabrication versatility.^[^ ^23^ ^]^ Numerous types of photocatalysts have been reported in the literature. These include photocatalysts like oxides such as TiO_2_, Fe_2_O_3_, Ag_3_PO_4_, CuO, ZnO, MoO_3_, and WO_3_, sulfides such as CdS, ZnInS_4_, nitrides such as g‐C_3_N_4_, and carbon‐based materials such as Graphene, nanotubes.^[^ ^17^ ^]^ In recent years, numerous studies have focused on researching and developing various photocatalysts to enhance their efficiency in photocatalytic hydrogen production. According to Liao et al. to obtain high photo conversion efficiency, it is crucial to ensure effective charge separation, prevent backward reactions of O_2_ and H_2_, and utilize both UV and visible light energy efficiently.^[^ ^3^ ^]^ An essential electrochemical property of the photocatalyst is its chemical stability against corrosion and photo‐corrosion when exposed to an aqueous solution.^[^ ^14^ ^]^

Titanium dioxide (TiO_2_) is one of the most promising photocatalysts due to its excellent properties(discussed in Table 2) leading to enhanced photocatalytic performance.^[^ ^25^ ^]^ TiO_2_ exists in three different crystal polymorphs/phases such as anatase, rutile, and brookite; among these phases, rutile is the most stable phase.^[^ ^26^ ^]^ The properties of the three phases are given in **Table** **1**. However, due to very high energy bandgap of TiO_2_ (3.0–3.2 eV), it results in absorption of only UV light and hence low photo‐conversion efficiency.^[^ ^4^ ^]^ Nevertheless, several studies have revealed that TiO_2_ has structural and chemical properties that allow the modification of bandgap and other properties.^[^ ^6^ ^]^ Consequently, research has focused on improving the properties of TiO_2_ photocatalyst to decrease its bandgap and enhance the photoconversion efficiency. A commercial TiO_2_ known as TiO_2_ P25 Degussa comprising a mixture of anatase and rutile phase with an 80:20 ratio, is widely utilized and studied due to its high photocatalytic activity.^[^ ^27^ ^]^ The benefits and drawbacks of TiO_2_ photocatalyst are given in **Table** **2**.

**Table 1:** Properties of TiO_2_ polymorphs.^[^ ^26^ ^]^

Property	Anatase	Rutile	Brookite
Crystal structure	Tetragonal	Tetragonal	Orthorhombic
Atoms per unit cell	4	2	8
Density, g cm^−3^	3.83	4.24	4.17
Band gas, eV	3.26	3.05	
Refractive index	2.57	2.95	2.81

**Table 2:** Advantages and Disadvantages of TiO_2_ photocatalyst in water splitting.^[^ ^21^ ^]^

Advantages	Disadvantages
Enhanced photo‐chemical stability Renewable hydrogen production through solar energy. Excellent resistance to photo‐corrosion Abundant, cheap, and non‐toxic Readily synthesized in nanocrystalline form through simple methods	Rapid electron‐hole pair recombination and potential backward reaction leading to formation of water. Wide band gap Significant over potential required for hydrogen production on the surface of TiO_2_.

Many other oxide catalysts have been reported in the literature, but they have their limits contributing to low photocatalytic performance. For instance, ZnO, CuO, Fe_2_O_3_, and SnO, exhibit poor photochemical stability in solution whereas other catalysts are comparatively expensive and challenging to acquire.^[^ ^28^ ^]^ According to Ahmad et al. most metal sulphide photocatalysts have a problem of photo corrosion but much of the recent literature suggests that a lot of modified sulfide photocatalysts tend to be good photocatalysts for hydrogen generation.^[^ ^21^ ^]^ Gandía et al. reported that majority of the photocatalysts exhibit activity only under UV light, with very few catalysts developed to be active under visible light.^[^ ^16^ ^]^

According to Villa et al., present photocatalytic solar to hydrogen (STH) efficiencies are reaching 1%, however, they are still lower than the efficiencies achieved by other well established hydrogen production methods based on fossil fuels.^[^ ^29^ ^]^ Saddique et al. reported that the overall performance of the photocatalytic hydrogen production process relies on the photocatalyst.^[^ ^14^ ^]^ Therefore, the advancements of effective photocatalyst systems is crucial to meet the requirements of solar to hydrogen efficiency and to advance photo‐catalytic hydrogen production toward practical applications.^[^ ^14^, ^29^ ^]^ A considerable amount of literature has been published on photocatalysts, however, the search for better photocatalysts continues to enhance the feasibility and sustainability of PWS as a method for hydrogen production.

### 3.2 Modification of TiO_2_ Photocatalyst

The research to date has focused on enhancing the photo‐catalytic activity of TiO_2_ catalysts under visible light conditions. The following modified TiO_2_ photocatalysts have shown increased photoactivity as a result of the modification to their structural and chemical properties.

#### 3.2.1 Metal‐Modified TiO_2_

One of the most widely used modification methods is the deposition of metals on to the surface of TiO_2_ catalyst. Metal dopants generally used include transition metals, noble metals, and rare‐earth metals.^[^ ^30^ ^]^ Liao et al. conclusively reported that the loading of metals such as platinum, gold, palladium, rhodium, nickel, copper, and silver on the TiO_2_ surface increases its photocatalytic activity.^[^ ^3^ ^]^ The loading of metal prevents the electron‐hole pairs recombination hence improving the hydrogen emission rate.^[^ ^17^ ^]^ This is supported by Rusinque et al. who observed that the volume of hydrogen produced using Pd‐doped TiO_2_ is approximately three times higher than that produced with unmodified TiO_2_.^[^ ^31^ ^]^ According to Hanaor et al., the metal dopants reduce the wide band gap of TiO_2_ and can either facilitate or inhibit the phase transformation from anatase to rutile, thereby affecting the photocatalytic activity and hydrogen production rate which are dependent on the structural configuration of TiO_2_.^[^ ^32^ ^]^

The introduction of metal dopants also causes a notable shift in absorption toward the visible light region.^[^ ^10^ ^]^ This aligns with the findings of the study conducted by Díaz et al., which illustrated that under both UV and visible light irradiation, the hydrogen production rate obtained with Cu/TiO_2_ was significantly greater than that with undoped TiO_2_.^[^ ^33^ ^]^ The increased photocatalytic activity upon exposure to visible light irradiation indicates that the metal doping reduces the wide energy bandgap of the TiO_2_ catalyst. Díaz et al. stated that the hydrogen production rate is influenced by the experimental configuration and cannot be utilized to compare the findings of various researchers.^[^ ^33^ ^]^ Nevertheless, it is beneficial to contrast the experimental outcomes of a novel or modified photocatalyst with those attained using a widely recognized commercial catalyst like TiO_2_ P25.

Generally, doping with Ag and Au is thermodynamically more stable compared to doping with Pt, Pd, and Ru.^[^ ^6^ ^]^ However, among all these metals, Platinum is the most extensively researched dopant due to its ability to yield a substantial rate of hydrogen production. However, the use of noble metal modified TiO_2_ catalysts for large‐scale hydrogen production is not economically feasible because of the expense and rarity of the noble metals.^[^ ^33^ ^]^ This has motivated the investigation of alternative co‐catalysts that are more cost‐effective.^[^ ^34^ ^]^

Based on the results collected by Sangpour et al., Adamu et al. noted that the photocatalytic performance of copper‐doped TiO_2_ is comparable to that of the Platinum doped TiO_2_.^[^ ^35^ ^]^ This aligns with the conclusion drawn by Díaz et al. who suggested that Copper is a promising substitute for Platinum as it is a cost‐effective, efficient co‐catalyst and yields a comparable H_2_ rate.^[^ ^33^ ^]^ In a theoretical investigation conducted by Hussein et al. to find the impact of copper doping on the photocatalytic performance of anatase TiO_2_, it was noted that the improved H_2_ rate using Cu/TiO_2_ was due to the reduced bandgap and increased charge transfer rather than the surface chemistry of the adsorbed water.^[^ ^36^ ^]^ Various studies have shown that in Cu/TiO_2_ catalysts, Cu exists in many different forms such as CuO (Cu(II)), Cu_2_O (Cu(I)), and Cu (Cu(0)).^[^ ^26^ ^]^ Adamu et al. found that the distribution of Cu(II) and Cu(I) was influenced by the pH used during the synthesis process.^[^ ^26^ ^]^

Numerous studies have extensively explored Cu‐based TiO_2_ catalyst and its application in photocatalytic water splitting. Hinojosa‐Reyes et al reported that hydrogen production through Cu‐loaded TiO_2_ was four times greater than bare TiO_2_.^[^ ^37^ ^]^ Chen et al. investigated Cu/TiO_2_ nanorod photocatalyst and observed that a 0.1wt%‐Cu/TiO_2_ catalyst resulted in efficient hydrogen production (1023.8 µmol h^−1^), which is ≈20 times higher than that resulted from pure TiO_2_ (49.4 µmol h^−1^).^[^ ^38^ ^]^ Notably, this rate approached that of Pt/TiO_2_ (1161.7 µmol h^−1^).^[^ ^38^ ^]^ In a recent investigation by Quyen et al., Cu‐TiO_2_ demonstrated excellent stability and resulted in a fairly constant hydrogen production rate even after being reused for five consecutive cycles.^[^ ^39^ ^]^ This indicates that the reusability and stability of the Cu/TiO_2_ catalyst make it a suitable candidate for industrial photocatalytic water splitting.

Another metal‐loaded TiO_2_ catalyst that has gained much attention due to its surface plasmonic resonance (SPR) effect is the Ni‐loaded TiO_2_ catalyst.^[^ ^40^ ^]^ Chen et al. reported that Nickel species such as metal nickel, nickel oxide, and nickel hydroxide are more cost‐effective compared to noble metals and have been proven to improve photocatalytic hydrogen production.^[^ ^41^ ^]^ Chen et al. also reported that Nickel is one the most suitable transition metals co‐catalysts due to its affordability, abundance, and high work function (Ni ∅ = 5.3 eV).^[^ ^42^ ^]^ Díaz et al. investigated various metal‐loaded TiO_2_ (M/TiO_2_) catalysts and found that the Ni/TiO_2_ catalysts demonstrated notable photocatalytic activity under UV irradiation.^[^ ^33^ ^]^ However, the H_2_ rate obtained with Cu/TiO_2_ photocatalyst was higher compared to that with Ni/TiO_2_ photocatalyst. This observation is consistent with the findings of Montoya et al. who observed that 1wt%Cu/TiO_2_ resulted in 84.7 µmol h^−1^ of hydrogen as compared to 1wt%Ni‐TiO_2_ which resulted in 33.9 µmol h^−1^ of hydrogen under similar conditions.^[^ ^43^ ^]^ Notably, there is limited research available on the utilization of Ni/TiO_2_ photocatalyst for the process of water splitting.^[^ ^42^ ^]^

**Figure 4:**
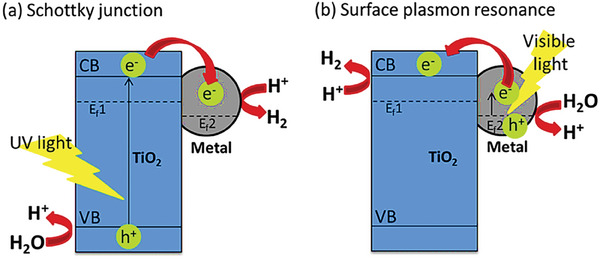
Photocatalytic water splitting on metal‐doped TiO_2_. Reproduced with permission.^[^ ^18^ ^]^ Copyright 2021, Elsevier.

The increased photocatalytic activity of metal‐doped TiO_2_ can be attributed to two phenomena such as a) Schottky junction under UV light irradiation and b) Surface plasmon resonance (SPR) effect under visible light irradiation. Schottky junction is established by the arrangement of bands at the metal‐semiconductor heterojunction which creates an electronic barrier, this enables transfer and capture of the electrons in to the metal, thereby reducing the charge recombination and ultimately enhancing the efficiency.^[^ ^17^ ^]^ The efficiency of the photocatalyst is affected by the metal's work function, representing the energy required to transition an electron from the Fermi level into the vacuum. The formation of large Schottky barrier results in enhanced hydrogen production as it enhances the disparity in work function between the metal (s) and the semiconductor (**Figure** **4**a).^[^ ^18^ ^]^ The SPR effect is depicted in Figure 4b. During exposure to visible light, photogenerated electrons in the localized surface plasmon resonance (LSPR) of metal can effectively move to the conduction band (CB) of TiO_2_ which undergo reductions at the catalyst's surface, thereby increasing the photocatalytic activity.^[^ ^18^ ^]^

#### 3.2.2 Bimetallic TiO_2_

Another interesting modification technique involves deposing bimetallic co‐catalysts on TiO_2_ and these types of catalysts have been extensively studied and widely utilized in industrial applications.^[^ ^10^ ^]^ Naseri et al. proposed that bimetallic deposited TiO_2_ leads to an enhanced hydrogen production rate compared to monometallic deposited TiO_2_.^[^ ^44^ ^]^ This corresponds with the findings of Tian et al. who reported that the bimetallic cocatalyst has the potential to serve as an effective cocatalyst for TiO_2_, enhancing its photocatalytic activity more efficiently.^[^ ^45^ ^]^ The literature contains numerous studies on bimetallic‐deposited TiO_2_ photocatalysts. Fuentes et al. studied the performance of Pt‐Ru, Pt‐Ir, Pt‐Ru‐Ir, and Ir‐Ru bi/tri metallic supported TiO_2_ catalyst in an oxygen evolution reaction (OER).^[^ ^46^ ^]^ Bimetallic catalysts have also been used in thermo‐catalytic processes such as methane reforming, carbon dioxide hydrogenation, and methane cracking.^[^ ^40^ ^]^

Bimetallic co‐catalysts composed of Cu and Ni, when doped on to various semiconductor materials, are known to enhance the efficiency of various valuable industrial reactions.^[^ ^47^ ^]^ Tian et al. investigated the Cu‐Ni/TiO_2_‐based photocatalytic system in water splitting.^[^ ^45^ ^]^ It was found that the Cu‐Ni bimetallic deposits function as active sites and result in high photocatalytic activity. In a recent investigation by M. et al. on Cu‐Ni/TiO_2_ for water splitting, it was observed that the Cu‐Ni alloy nanoparticles exhibited substantial photo reactivity under solar light, leading to a H_2_ rate of 35.4 mmol g^−1^.h^−1^.^[^ ^40^ ^]^ The study concluded that this effect was attributed to surface plasmon resonance (SPR) and the synergistic effect between Cu and Ni metal species, ultimately enhancing the photocatalytic activity.^[^ ^40^ ^]^

A highly dispersed and novel 1wt% Cu:Ni/TiO_2_ catalyst synthesized by Ibrahim et al. resulted in maximum H_2_ production rate of 8.5 mmol h^−1^.g^−1^.^[^ ^48^ ^]^ Majeed et al. synthesized 0.8Cu(OH)_2_‐0.2 Ni(OH)_2_/ TiO_2_ nanorods and achieved a maximum H_2_ production rate of 35 mmol h^−1^.g^−1^.^[^ ^49^ ^]^ Nevertheless, these catalysts were only evaluated under UV light source and their activity under visible light was not assessed.^[^ ^48^, ^49^ ^]^ Mohd Amin et al. noted that the enhanced hydrogen production rate resulted using bimetallic catalyst was due to the reduced energy band gap of the catalyst.^[^ ^9^ ^]^ Other bimetallic catalysts that have been developed are given in **Table** **3**. Various studies investigating bimetallic catalysts for PWS have examined the impact of different factors such as synthesis conditions, metals ratio and concentration, dosage of catalyst, and concentration of sacrificial reagent. However, there have been relatively few studies examining the impact of different sacrificial reagents or electron donors on the rate of hydrogen production using bimetallic catalysts.

**Table 3:** Modified TiO_2_ catalysts for photocatalytic water splitting.

Photocatalyst	Metal concentration [wt%]	Amount of catalyst	Light source	Sacrificial reagent [Vol%]	H_2_ production rate [mmol time^−1^ g^−1^ Catalyst]	Quantum efficiency/AQY	Ref	Year
Metal modified TiO_2_								
Pt/TiO_2_ (P25)	0.3%	50 mg	LED UV light	Methanol(20%)	58 mmol g^−1^ h^−1^		[11]	2023
Pd/TiO_2_	1%	0.0065 g/ 20 mL	200 W SB‐1000P/F	1,2‐ethanediol	44.5 mmol g^−1^ h^−1^		[58]	2015
Au/TiO_2_ (P25)	2%	6.5 mg	100 W SB‐100P/F	Glycerol (10%)	30.3 mmol g^−1^ h^−1^		[59]	2018
Ag/TiO_2_ (P25)	1.5%	–	254 nm UV light	–	23.5 mmol g^−1^ h^−1^	>19%	[60]	2019
Fe/TiO_2_ (P25)	2%	0.2 g/200 mL	300 W Halogen Visible lamp	Methanol(25%)	<10 mmol g^−1^ h^−1^		[33]	2021
Cu/TiO_2_ (P25)	1.5%	0.2 g/200 mL	300 W Synergy UV lamp	Methanol(25%)	5100 µmol g^−1^h^−1^		[33]	2021
Cu/TiO_2_	–	0.01 g/1000 mL	300 W Xenon lamp	methanol(10%)	45.6 mmol h^−1^		[39]	2021
Ni/TiO_2_ (P25)	5%	0.1 g/100 mL	300 W Xenon Lamp	methanol (30%)	600 µmol h^−1^		[41]	2018
Ni/TiO_2_ (P25)	1%	10 mg / 50 mL	450 W Hg lamp	Methanol (50%)	3390 µmol g^−1^ h^−1^	2.8%	[43]	2018
Co‐TiO_2_	1%	10 mg / 50 mL	450 W Hg lamp	Methanol (50%)	24.8 µmol h^−1^	2.1%	[43]	2018
Non‐metal modified TiO_2_								
N‐TiO_2_ films	–	–	Xenon Lamp	Methanol (10%)	4500 µmol h ^−1^ m^−2^		[51]	2013
S‐TiO_2_		100 mg/100 mL	AM 1.5 solar simulator	Methanol (20%)	163.9 µmol g^−1^ h^−1^		[61]	2016
F‐TiO_2_			300 W Xenon lamp	–	18 270 µmol g^−1^ h^−1^	21.6%	[61]	2018
Co‐doped TiO_2_ (Non‐metallic, metal‐metal, Non‐metal, and metal co‐doping)								
Pt‐Pd/ Nb‐TiO_2_	3% NbPtPd (1:1)	0.5 g L^−1^	500 W Hg‐Xenon lamp	Methanol (30%)	0.8 mmol g^−1^ h^−1^ (visible) 4.5 mmol g^−1^ h^−1^ (UV)	3% UV, 0.8% Visible	[62]	2018
Fe/Ni‐TiO_2_	5% Fe, 4% Ni	0.4 g/600 mL	500 W Xenon Lamp	Methanol (10%)	361.64 µmol g^−1^ h^−1^		[63]	2012
Ni/N‐TiO_2_ (Nanotubes)	10% Ni	150 mL	Mercury lamp	Glycerol (10%)	30 973 µmol m^−2^		[64]	2020
Ni‐NiO‐TiO_2_	1%	100 mg/347.8 mL	5 W UV‐LED solo P lamp	Methanol (50%)	1600 µmol g^−1^		[65]	2023
Cu‐Ni/TiO_2_	1% (1:1)	80 mg/80 mL	300 W Xenon lamp	Methanol (37.5%)	13.5 mmol g^−1^ h^−1^		[45]	2014
N‐Ni/C/TiO_2_	15%	50 mg/100 mL	2000 W Mercury lamp	Methanol (25%)	0.383 mmol s^−1^.g^−1^		[66]	2017
Ni‐Pd/TiO_2_	1Ni1Pd10	50 mL /5 mL	400 W Mercury lamp	Methanol (50%)	210 µmol/h		[67]	2017
Semiconductor coupled TiO_2_								
ZnO‐TiO_2_ (hollow spheres)		100 mg/100 mL	300 W Xenon lamp	Methanol (20%)	0.152 mmol g^−1^ h^−1^		[68]	2017
WSe_2_‐TiO_2_ (WSe_2_ nanosheets)	20% WASe_2_	10 mg/30 mL	Xenon lamp	Methanol (17%)	2.28 mmol g^−1^ h^−1^	AQY = 43.8%	[69]	2022
SnO_2_/TiO_2_ (TiO_2_ nanosheets)		20 mg/100 mL	300 W Xenon lamp	Methanol (20%)	16.7 mmol g^−1^ h^−1^		[70]	2022
WO_3_‐TiO_2_ (Nanofibers)	5%	30 mg/60 mL	100 W Mercury lamp	Na_2_S/Na_2_SO_3_	107.15 µmol g^−1^ h^−1^		[71]	2020
Ternary TiO_2_								
Pt/TiO_2_‐ZnO	0.5% Pt	0.5 g/250 mL	400 W Mercury lamp (UV cut filter)	Methanol (10%)	2150 µmol g^−1^ h^−1^		[55]	2016
Pd/TiO_2_‐WO_3_ (TiO_2_ nanotubes)	0.013% Pd	500 ppm/0.6 L (Solar) and 500 ppm/0.25 L(UVA)	250 W/m^2^‐solar simulated light and 4 lamps of 15 W‐UVA light	Methanol (50%)	Solar: 5.3 × 10^−5^ mol min^−1^ g^−1^ UVA: 5.3 × 10^−5^ mol min^−1^ g^−1^	2.3% (solar) 7.7% (UVA)	[57]	2018
TiO_2_/Ti_3_C_2_/g‐C_3_N_4_		30 mg	300 W Xenon lamp	Triethanolamine (10%)	1150 µmol g^−1^ h^−1^		[72]	2021

Table 3 presents metal‐doped, non‐metal doped, and bimetallic TiO_2_ catalysts that have been researched in the past few years. From Table 3, it can be seen that most studies on metal/nonmetal/bimetallic doped TiO_2_ catalysts were conducted using a well‐known commercial TiO_2_ (P25 TiO_2_) catalyst. Interestingly, in their study, Adamu et al. examined the effects of various synthesis methods on the properties of Cu/TiO_2_ catalyst for the reduction of carbon dioxide.^[^ ^26^ ^]^ The results indicated that doping P25 TiO_2_ (80% anatase, 20% rutile) with Cu using the incipient wetness impregnation method resulted in a decrease in the anatase phase to 73% while the rutile phase remained unchanged. Conversely, copper doping using the sol‐gel method prevented the transition of the anatase to the rutile phase and increased the anatase to rutile ratio (A/R).^[^ ^26^ ^]^ There are various studies conducted on metal‐doped TiO_2_ catalysts for photocatalytic water splitting. However, several studies reviewed did not consider the impact of doping on the A/R ratio and its subsequent effect on photocatalytic hydrogen production.

To summarize, doping TiO_2_ with metals effectively reduces the rate of charge recombination, improves response to visible light, and hence increases the activity of TiO_2_ in photocatalytic hydrogen production. However, Yang et al. stated that the type and quantity of dopants are crucial parameters that influence the photocatalytic performance such that if the amount of dopant surpasses a certain concentration, the lattice structure of TiO_2_ becomes significantly distorted, consequently restricting improvements in photocatalytic performance.^[^ ^30^ ^]^ Therefore, many researchers have studied the impact of metal concentration in the photocatalyst on its activity. For instance, Díaz et al. studied the effect of Cu loading on TiO_2_ on the rate of hydrogen production and found that as the concentration of Cu increased from 0.1 to 0.5 wt%, the H_2_ rate increased significantly; nevertheless, increasing Cu content above 3 wt% led to a decrease in H_2_ rate.^[^ ^33^ ^]^ Tian et al. found similar results and reported that the excessively elevated concentration of Cu/Ni alloy leads to reduced photocatalytic activity of Cu/Ni‐TiO_2_.^[^ ^45^ ^]^ The maximum hydrogen production rate was achieved when ratio of Cu to Ni was equimolar; furthermore, increasing the metal content beyond this ratio resulted in a decreased hydrogen production rate.^[^ ^45^ ^]^ Therefore, it is crucial to determine the optimal metal content in metal‐loaded TiO_2_ catalysts that maximizes the rate of hydrogen production.

#### 3.2.3 Non‐Metal Modified TiO_2_

Previous research findings show that non‐metal‐modified TiO_2_ photocatalysts also improve the photocatalytic activity of TiO_2_. Various non‐metals such as C, F, S, and N have been reported to increase the photocatalytic activity of TiO_2_.^[^ ^3^ ^]^ Eidsvåg et al. reported the primary roles of the dopants are to increase the light absorption intensity and the ability to absorb visible light.^[^ ^6^ ^]^ The loading of non‐metals on TiO_2_ reduces the energy band gap which increases the visible light photocatalytic performance.^[^ ^17^ ^]^ Among all these non‐metals, nitrogen‐doped TiO_2_ is the most studied photocatalyst and it also results in high hydrogen production rates.^[^ ^25^ ^]^ Wang et al. discovered that the N/TiO_2_ films resulted in a hydrogen rate of 601 µmol g^−1^.h^−1^ significantly surpassing the rates obtained with pure TiO_2_ films and undoped P25 TiO_2_.^[^ ^50^ ^]^ Wang et al. in another study found that the rate of hydrogen production was 760 µmolH_2_.h^−1^.m^−2^ when pure TiO_2_ films were used, which increased to 4500 µmolH_2_.h^−1^.m^−2^ when N‐doped TiO_2_ films were used.^[^ ^51^ ^]^ This indicates that the non‐metal dopant significantly enhanced the efficiency of TiO_2_. Non‐metals reduce both the band gap energy, formation of recombined charge centers, and result in increased response to visible light irradiations; hence non‐metal doping is another important strategy that can be used to enhance hydrogen production.^[^ ^18^ ^]^ Interestingly, Yang et al. reported that non‐metal is more efficient than metal modification in enhancing the photocatalytic performance of TiO_2_.^[^ ^30^ ^]^ Nevertheless, a study focused on comparative analysis between metal and mom‐metal doped TiO_2_ catalysts for efficient hydrogen production was not found in the literature.

#### 3.2.4 Semiconductor Coupling TiO_2_

A review of the literature shows that the performance of pure TiO_2_ for photocatalytic hydrogen production is extremely low under visible light irradiation. Interestingly, Ahmad et al. highlighted in a review that comparative studies have shown that heterojunction photocatalysts result in better photocatalytic activity as compared to single photo‐catalysts.^[^ ^21^ ^]^ Many recent studies of modified TiO_2_ catalysts coupled with binary composites (e.g., WO_3_, SiO_2_, Al_2_O_3_, SnO_2_, CdS, PBS, Bi_2_S_3_) and transition metals oxides (e.g., Cu_2_O, Fe_2_O_3_, ZnO, NiO) have demonstrated improvement in the performance of TiO_2_ photocatalyst.^[^ ^17^ ^]^ Combining TiO_2_ with other materials leads to enhanced surface area and visible light absorption.^[^ ^21^ ^]^ The coupling of non‐oxide photocatalysts with oxide or other non‐oxide photocatalysts forms a heterojunction. Li et al. reported that the five types of heterojunctions are i) convention type‐II heterojunction, ii) p‐n type heterojunction, iii) surface heterojunction, iv) direct Z‐scheme heterojunctions, and iv) semiconductor‐graphene heterojunction.^[^ ^52^ ^]^ The formation of heterojunction improves the photocatalytic activity by combining a semiconductor with a larger band gap with semiconductor having a smaller band gap. This improves functionality under visible light and enhances charge separation efficiency.^[^ ^21^ ^]^

Lai et al. investigated TiO_2_ photocatalyst coupled with WO_3_ and found that it results in enhanced electron‐hole separation and visible light response.^[^ ^53^ ^]^ Similarly, the study by Georgieva et al. demonstrated that the coupled TiO_2_‐WO_3_ heterojunction increases photocatalytic activity because of the reduced electron‐hole recombination.^[^ ^54^ ^]^ Several studies have revealed that other metal oxides, sulfides, and nitrides have been effective as co‐catalysts with TiO_2_ to improve its photocatalytic activity.^[^ ^17^ ^]^ Various other heterojunction photocatalysts reported include CdS/TiO_2_, CoSe/TiO_2_, SnO_2_/TiO_2_, ZrO_2_/TiO_2_, Ag_2_S/TiO_2_, Fe_3_O_4_/TiO_2_, Bi_2_O_3_/TiO_2_ and SiO_2_/TiO_2_ etc. ^[^ ^30^ ^]^ All these photocatalysts demonstrated a shift in absorption spectrum of the TiO_2_ photocatalyst toward the visible light region resulting in increased photo conversion efficiency.^[^ ^30^ ^]^ The heterojunction formed between non‐oxide and oxide or other non‐oxide photocatalysts enhances the photocatalytic activity; however, many of the non‐oxide composite photocatalysts encounter the issues of photo corrosion in aqueous solutions which limits their application in water splitting.^[^ ^3^ ^]^

#### 3.2.5 Ternary TiO_2_

Ternary photocatalytic systems are composed of three different semiconductors. Xie et al. investigated the ternary system of Pt/TiO_2_‐ZnO (Ti/Zn = 10) for photocatalytic hydrogen production and the data obtained showed that the maximum H_2_ rate was 2150 µmol h^−1^ g^−1^ and the stability of the photocatalyst also improved.^[^ ^55^ ^]^ Fajrina et al. reported that according to most of the literature produced on ternary TiO_2_ systems, most ternary composites contain heterojunctions of two semiconductors deposited with metal.^[^ ^17^ ^]^ For example, Spanu et al investigated the ternary photocatalyst Pt/TiO_2_‐WO_3_ and found a hydrogen production rate of 5.2 µLH_2_.h^−1^.cm^−2^.^[^ ^56^ ^]^ Toledo Camacho et al. investigated Pd/TiO_2_‐WO_3_ and observed that the H_2_ rate was in the order Pd/TiO_2_(nanotubes)/WO_3_ >Pd/TiO_2_(P25)/WO_3_ > Pd/TiO_2_(P25).^[^ ^57^ ^]^ Other ternary TiO_2_‐based photocatalysts reported by Fajrina et al. include Cu/TiO_2_/Ti_3_C_2_, Pt/WO_3_/TiO_2_, Cu‐TiO_2_/porphyrin, and Pt‐RuO_2_‐TiO_2_ etc.^[^ ^17^ ^]^

Table 3 presents various types of modified TiO_2_ catalysts studied in recent years. However, the results obtained should not be compared with each other due to variations in experimental conditions.

## 4 Factors Affecting Photocatalytic Water Splitting

The efficiency of a photocatalyst and its ability to produce hydrogen during photocatalytic water splitting are influenced by several factors. By optimizing various parameters during the reaction, the photocatalyst's efficiency can be significantly improved. Hence, it is crucial to control these parameters to achieve high photoconversion efficiency and to enhance the total solar to hydrogen (STH) efficiency of the process. These factors include both the physical properties of the photocatalyst, such as its structure, morphology, band gap, corrosion resistance, as well as the experimental parameters like pH, temperature, the type and concentration of sacrificial reagent, and the intensity of the light. Therefore, it is important to understand and control these parameters to advance the performance of photocatalytic water splitting process.

### 4.1 Structure, Crystallinity and Morphology

The synthesis methods used for catalyst preparation affect the structure and morphology of the photocatalyst. The different reaction conditions used during the catalyst formation result in different crystal sizes, shapes, and structures.^[^ ^21^ ^]^ Adamu et al. stated that different crystal structures and orientations of the polymorphs of TiO_2_ result in distinct photocatalytic properties.^[^ ^26^ ^]^ For instance, Park et al. found that anatase phase exhibits higher photocatalytic activity compared to the rutile phase, this is due to the different structure of the two phases contributes to different energy band gaps.^[^ ^73^ ^]^ In general, anatase phase results in enhanced photocatalytic performance compared to other phases because of its appropriate band gap and higher kinetic stability.^[^ ^18^ ^]^ Interestingly, Eddy et al reported that the combination of TiO_2_ polymorphs in a binary mixture exhibited a notable enhancement in photocatalytic activity, with the binary mixture of anatase and rutile being extensively studied.^[^ ^27^ ^]^ Similarly, Eidsvåg et al. reported in a review that the optoelectronic properties and hence catalyst performance are influenced by its crystallinity, with highly crystalline catalysts outperforming their amorphous counterparts.^[^ ^6^ ^]^ Liu et al. investigated the crystalline TiO_2_ nanotubes and amorphous TiO_2_ nanotubes and concluded that the crystallite structure yielded better photocurrent properties due to the reduced charge recombination.^[^ ^74^ ^]^ This aligns with the findings of Fajrina et al. who reported that the smaller sized crystalline photocatalysts facilitates rapid transfer of e^−^ and h^+^ to the active sites, thereby reducing the charge recombination.^[^ ^17^ ^]^

Anggoro et al. stated that rate of hydrogen generation is significantly influenced by the morphology of TiO_2_ nanoparticles indicating that morphological modification is an efficient method to enhance the photocatalytic performance.^[^ ^28^ ^]^ In a review, Jagadeesh Babu et al. highlighted that semiconducting nanostructure in one‐dimensional (1D: Nanorods, nanowires, nanotubes) and two‐dimensional (2D: nanosheets, nanolayers, nanofibers) forms exhibit enhanced photocatalytic activity due to increased charge separation and reduced recombination rate.^[^ ^75^ ^]^ Various 1D, 2D, and 3D TiO_2_ structures investigated for photocatalytic water splitting are given in **Table** **4**. Wang et al. investigated the photocatalytic performance of NiO‐TiO_2_ nanorod structure for hydrogen production using a methanol‐water mixture and found that the hydrogen production rate was 1.3 higher with 2NiO‐TiO_2_ catalyst compared to pure TiO_2_.^[^ ^76^ ^]^

**Table 4:** Different TiO_2_‐based nanostructures reported for hydrogen production.

Catalyst	Structure	H_2_ rate	Reference
TiO_2_	Nanosheets	270 µmol h^−1^	[77]
0.18%‐Pd/TiO_2_	Nanosheets	3096 µmol g^−1^ h^−1^	[78]
2NiO/TiO_2_	Nanorods	701 µmol g ^−1^cat^−1^	[76]
Pd0.22Pt0.78‐TiO_2_	Nanowires	11 mmol g ^−1^cat^−1^	[79]
CoNi‐TiO_2_	Nanoflowers	6580.9 µmol g^−1^ h^−1^	[80]

The properties of the catalyst such as shape and composition depends on the temperature utilized for catalyst preparation which is determined by the synthesis method used.^[^ ^17^ ^]^ Buraso et al. studied how varying the calcination temperature affects the photocatalytic performance of TiO_2_ catalyst in degradation of methyl orange.^[^ ^81^ ^]^ It was observed that as the calcination temperature increased, the particle size and degree of crystallinity of the TiO_2_ catalyst also increased. It was also observed that with an increase in calcination temperature from 400 to 700 °C, the TiO_2_ crystal system changed from anatase to rutile and the direct band gap (Eg) decreased from 3.30 to 2.98 eV. In summary, it was determined that the improved activity of the TiO_2_ was attributed the purity of the anatase phase, reduced particle size, and increased surface area of nanoparticles.^[^ ^81^ ^]^ This is in agreement with Eidsvåg et al. who reported that the size of nanomaterials and cocatalysts can influence the photocatalytic activity, with smaller particles being more favorable to reduced electron‐hole recombination probability.^[^ ^6^ ^]^

Anggoro et al. reported that the morphology and structure of mono or bimetallic catalysts can exist in different forms such as alloys, core‐shell, and Janus type.^[^ ^28^ ^]^ Tian et al. and Kotesh Kumar et al. synthesized Cu/Ni‐TiO_2_ photocatalyst using a simple hydrothermal process and co‐impregnation method, respectively.^[^ ^45^ ^]^ Both approaches led to the formation of Cu‐Ni alloy on TiO_2_ nanoparticles and the synergetic effect between the two metals resulted in decreased electron‐hole recombination rates which in turn enhanced the hydrogen production rate. Ramírez et al. investigated CuO/TiO_2_ and NiO/TiO_2_ core‐shell catalysts for photocatalytic production of hydrogen and found that CuO/TiO_2_ resulted in the maximum H_2_ rate of 153.8 µmol g^−1^ h^−1^ which was 3.2 and 11.2 and times higher than that resulted from NiO/TiO_2_ and TiO_2_ P25, respectively.^[^ ^82^ ^]^ The core‐shell structure resulted in creation of a heterojunction between the TiO_2_ shell and CuO core which inhibited the recombination of electrons and holes and increased charge transfer resulting in increased hydrogen production rates.

Overall, this indicates that the photocatalytic efficiency of TiO_2_ is strongly determined by its particle size, crystallite size, shape, and morphology hence, the selection of appropriate synthesis methods is vital for enhanced hydrogen production rate. Various synthesis methods have been reviewed in Section 5.

### 4.2 Band Gap

**Figure 5:**
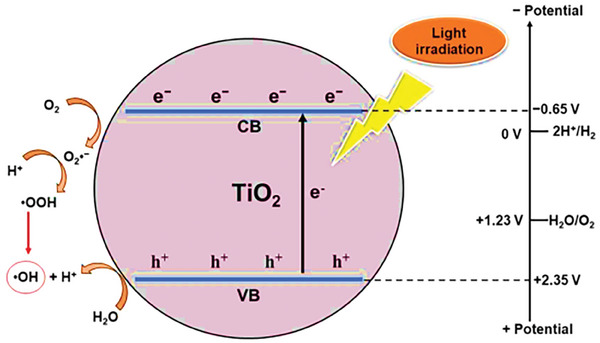
Schematic diagram of photocatalytic process and TiO_2_ band gap. Reproduced under the terms of the CC BY 4.0 license.^[^ ^27^ ^]^ Copyright 2023, Copyright MDPI.

The bandgap is the most crucial characteristic of the photocatalysts representing the energy required for an electron to transition from the valence band (VB) to the conduction band (CB)^[^ ^6^ ^]^ For water splitting reaction, the semiconductor's conduction band must exhibit a more. negative potential than the redox potential of H+/H_2_, while its valence band must possess a more positive potential than the redox potential of O_2_/H_2_O; this necessitates that the acceptor's relative potential is thermodynamically lower than the conduction band of the semiconductor.^[^ ^17^ ^]^ N. Ain et al. reported that the band gap of TiO_2_ is suitable for water splitting, as the top of the VB (+2.7 V versus NHE at pH 7) is more positive compared to O_2_/H_2_O redox couple (+1.23 V versus NHE at pH 7) and the bottom of the CB (−0.5 V versus NHE at pH 7) is more negative than the H^+^/H_2_ redox couple (0 V versus NHE at pH 7).^[^ ^9^ ^]^ Eidsvåg et al. reported that for photocatalytic water splitting, a photocatalyst with a bandgap of at least 1.23 eV is necessary, as depicted in **Figure** **5**.^[^ ^6^ ^]^

Notably, with a rise in the band gap of the photocatalyst, it absorbs light with shorter wavelengths, indicating that photocatalysts with bandgap exceeding 3.15 eV solely absorb UV light and not visible light.^[^ ^18^ ^]^ However, the band energy gap of TiO_2_ is 3.2 eV which prevents it from effectively catalyzing the water‐splitting reaction in the presence of visible light irradiation.^[^ ^21^ ^]^ Hence, to enable the visible light absorption, it is essential to use effective strategies discussed in section 3.2 to further reduce its band gap.^[^ ^17^ ^]^ Díaz et al. demonstrated that 0.5 wt% Cu/TiO_2_ resulted in a bang gap value of 3.03 eV.^[^ ^33^ ^]^ The results obtained by M. et al. for single and dual metal‐doped TiO_2_ photocatalysts are given in **Table** **5**.^[^ ^41^ ^]^

**Table 5:** Band gap and H_2_ rate of mono(Cu‐TiO_2_, Ni‐TiO_2_) and Bimetallic (Cu‐Ni/TiO_2_) photocatalysts.^[^ ^41^ ^]^

Photocatalyst	Band gap [eV]	H_2_ Uptake [µmol g^−1^cat^−1^]
TiO_2_	3.20	nd
2Cu/TiO_2_	2.92	180.9
5Ni/TiO_2_	2.78	253.0
0.5Cu‐5Ni/TiO_2_	2.83	nd
1Cu‐5Ni/TiO_2_	2.78	nd
2Cu‐5Ni/TiO_2_	2.70	634.5
3Cu‐5Ni/TiO_2_	2.64	nd

Table 5 shows how doping with different metals affects the energy band gap of TiO_2_, the results obtained for Cu/TiO_2_ agree with that by Diaz et al.^[^ ^33^ ^]^ It can be seen that dual metal doped TiO_2_ (3Cu‐5Ni/TiO_2_) decreases the band gap from 3.2 to 2.63 eV. It is notable that the quantity of metal loadings also impacts the band gap of the photocatalyst, hence its ability to absorb visible light irradiation.

### 4.3 Sacrificial Reagent/Electron Donor in Water Solution

**Figure 6:**
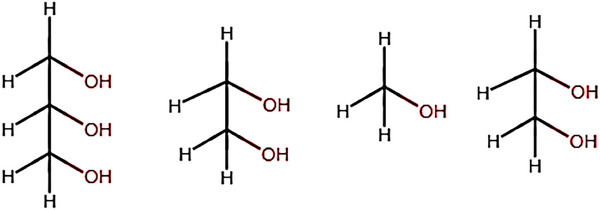
Structures of commonly used sacrificial reagents.^[^ ^17^ ^]^

Sacrificial agents are organic species that function as scavengers of holes and increase photocatalytic activity as compared to pure water.^[^ ^18^ ^]^ Sacrificial reagents are added to the water solution to prevent the rapid reverse reaction of hydrogen (H_2_) and oxygen (O_2_) to form water and they function as reducing agents increasing the hydrogen production rates.^[^ ^21^ ^]^ The most widely used sacrificial reagents include alcohols such as methanol, phenol, and glycerol; adding alcohols to improve the photocatalytic activity and hydrogen production rates is known as photo reforming.^[^ ^17^ ^]^ Chen et al. demonstrated that hydrogen production using different sacrificial reagents decreases in the following sequence: glycerol > ethylene glycol > methanol > ethanol.^[^ ^83^ ^]^ Based on these results Fajrina et al. indicated that the sacrificial reagent should contain an α‐H to OH groups as these α‐H are liberated into H^+^ ions which are converted into H_2_ using electrons during the photo‐reforming reactions (**Figure** **6**).^[^ ^17^ ^]^ This aligns with the findings of Qanugo et al. which stated that Glycerol contains the most α‐H hydrogen atoms compared to other alcohols thereby resulting in the highest H_2_ production rate.^[^ ^18^ ^]^ However, López et al. examined the impact of various sacrificial reagents on the hydrogen production rate using a Pt/TiO_2_ catalyst. They observed that the hydrogen production rate decreased in the sequence: methanol > ethanol > ethylene glycol > glycerol.^[^ ^84^ ^]^ This result contradicts with the findings by Chen et al which could be attributed to variations in reaction conditions.^[^ ^83^ ^]^ Kumaravel et al. investigated how different sacrificial reagents influences the performance of oxide photocatalysts and proposed that glucose and glycerol are the most efficient sacrificial reagents due to their accessibility, low toxicity, affordability, and easy dehydrogenation compared to other alcohols.^[^ ^7^ ^]^

Other studies have also considered the relationship between hydrogen production and concentration of the sacrificial reagents. Research findings of Lalitha et al. showed that hydrogen production increased up to five times with increased methanol concentration over a 1wt% Ag_2_O/TiO_2_ photocatalyst.^[^ ^85^ ^]^ Tian et al. investigated the impact of methanol concentration on the rate of H_2_ production over TiO_2_‐1%Cu/Ni (1:1) photocatalyst and observed that photocatalytic performance is notably reduced when the methanol concentration deviates from 37.5 vol%.^[^ ^45^ ^]^ This indicates that achieving enhanced hydrogen rates requires appropriate concentration of electron donors. Jiang et al. studied the impacts of mixed sacrificial reagents (single, binary, and ternary systems) on hydrogen production rate over TiO_2_ catalyst and discovered that the mixing of suitable sacrificial reagents in appropriate concentration results in enhanced photocatalytic activity and hydrogen production rate.^[^ ^86^ ^]^ However, as observed in Table 3, the most frequently used sacrificial reagent is methanol; this choice is likely influenced by its cost‐effectiveness and ready accessibility.

### 4.4 Intensity of Light

**Figure 7:**
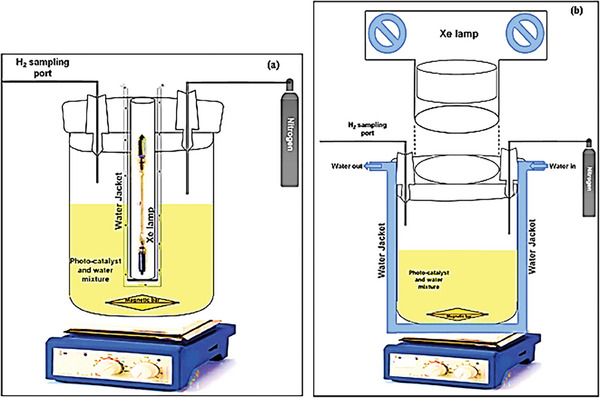
Type I and Type II photoreactor. ^[^Reproduced with permission.^[^ ^61^ ^]^ Copyright 2019, Elsevier.

One approach to enhance the photocatalytic hydrogen generation rate is to increase the intensity of light by using higher energy sources.^[^ ^18^ ^]^ Regarding UV photon flux, two regimes are identified for photocatalytic water splitting.^[^ ^23^ ^]^ These consist of a first‐order regime for fluxes at ≈25 mW cm^−2,^ typically used for laboratory research, and a half‐order regime at higher light intensities.^[^ ^21^ ^]^ In the first‐order regime, chemical reactions deplete the charges at a faster rate compared to recombination reactions, whereas in the half‐order regime, the recombination reaction usually controls the overall rate.^[^ ^17^ ^]^ Reim et al. examined the impact of varying light intensity on the rate of photocatalytic hydrogen production, and the findings indicated that higher light intensity or photon flux led to an increase in the amount of hydrogen produced.^[^ ^87^ ^]^ Similar results were reported by Fajrina et al. in a review.^[^ ^17^ ^]^ Wavelength and irradiation distribution across the reactors are two factors associated with the energy source that influence the photocatalytic efficiency. The light distribution across the reactor is determined by the position of the light source.^[^ ^88^ ^]^ Kumaravel et al. in a review reported that the maximum hydrogen yield was obtained in a reactor featuring an internal light irradiation source (Type I) as compared to a reactor with a light source placed outside at a particular distance (Type II) from the reactor; Type II allows the photocatalyst to directly absorb more photons in this configuration. The schematic of the two types of photoreactors is shown in **Figure** **7**.^[^ ^61^ ^]^

### 4.5 Temperature

According to Fajrina et al., thermodynamically, temperature cannot stimulate photocatalytic activity as it is not involved in the generation of e^−^ and h^+^; however, temperature indirectly increases the photocatalytic activity by enhancing the desorption of the formed product from the surface of the photocatalyst and hence increases the rate of the reaction.^[^ ^17^ ^]^ Both Ahmad et al. and Fajrina et al. stated that the operating temperature varies for different materials.^[^ ^17^, ^21^ ^]^ Although photocatalytic reactions are conducted at room temperature, increasing the temperature positively influences the activity of certain photocatalysts.^[^ ^89^ ^]^ Zhang et al. found that the ideal temperature range for the photocatalytic performance of crystalline TiO_2_ is 60–80 °C.^[^ ^90^ ^]^ Qanugo et al. reported that the higher temperature also results in higher electron transfers from the valence band to higher energy levels forming electron‐hole pairs which can initiate the oxidation and reduction reactions hence allowing reactions to complete more efficiently.^[^ ^18^ ^]^ Boudjemaa et al. observed that as the temperature increased from 30 to 50 °C, the H_2_ production rate increased from 59 to 370 mol g^−1^.s^−1^.^[^ ^91^ ^]^ Muscetta et al. studied the impact of temperature on the rate of hydrogen production with 1wt% Cu_2_O/TiO_2_ nanocomposite catalyst and observed that as the temperature rose from 20 to 80 °C, the hydrogen rate increased by 4.5 times.^[^ ^92^ ^]^ Velázquez et al. similarly stated that the rate of hydrogen production is significantly influenced by the reaction temperature.^[^ ^93^ ^]^ Interestingly, Kumaravel et al. emphasized the importance of maintaining the reaction temperature constant throughout the reaction because increased temperatures decrease the H_2_ production efficiency through decreased trapping of charge carriers and reduced adsorption of reactants on the catalyst surface.^[^ ^61^ ^]^

### 4.6 pH

Production of hydrogen from water is directly proportional to the concentration of protons which is determined by the pH of the solution.^[^ ^18^ ^]^ The pH of the solution also affects the stability and lifetime of a catalyst.^[^ ^4^ ^]^ Fajrina et al. reported that altering pH represent a method to shift the band gap energy of a photocatalyst.^[^ ^17^ ^]^ Ali Ghasemi reported that the efficiency of hydrogen production is higher in a weak basic solution than in an acidic or stronger basic solution (pH>10).^[^ ^24^ ^]^ Wu et al. also noted that CuOx/TiO_2_ photocatalyst resulted in a maximum H_2_ rate in a mild basic solution.^[^ ^94^ ^]^ The findings of many other studies, including the review conducted by Qanugo et al. indicated that TiO_2_ is unstable in strong acidic or basic solutions.^[^ ^18^ ^]^ However, NADA et al. found that greater amount of H_2_ was generated in an acidic medium compared to a basic medium, indicating that at acidic pH levels, photocatalyst exhibit greater adsorption of H^+^ ions, thereby enhancing the probability of reduction to produce H_2_.^[^ ^95^ ^]^ However, in a review Zhang et al. concluded that according to prior studies, photocatalytic reactions in basic solution generally offer more benefits for improving H_2_ generation.^[^ ^90^ ^]^ Karimi Estahbanati et al. performed a kinetic analysis to assess the impact of pH on the rate of photocatalytic H_2_ production and observed that the highest rate was achieved at a pH≈8 for all the substrate or sacrificial reagents investigated.^[^ ^96^ ^]^

Several studies also pointed out the effects of other factors on photocatalytic activity and hydrogen production rates. These factors include corrosion resistance, oxygen vacancies, and operating pressure, stirring rate of the reactor solution.

## 5 Synthesis Methods

As discussed in section 4.1, photocatalytic H_2_ production also depends on the composition, morphology, and surface properties of the photocatalysts, and these properties are influenced by the methods used to synthesize or modify the catalysts. For example, Chakhtouna et al. found that the photocatalytic activity of Ag doped TiO_2_ nanoparticles is notably impacted by the synthesis parameters and the methods used.^[^ ^97^ ^]^ Adamu et al. reported that various forms (e.g., crystals, nanoparticles, powder) of single or mixed‐phase TiO_2_ can be obtained based on the synthesis method utilized.^[^ ^26^ ^]^ According to Eidsvåg et al., the choice of method for doping or modifying TiO_2_ with metals affects its performance.^[^ ^6^ ^]^ Saleh et al. studied the influence of co‐catalyst loading method on the rate of hydrogen generation; the results obtained are presented in **Table** **6**.^[^ ^11^ ^]^

**Table 6:** Effect of metal loading methods on hydrogen production rate.^[^ ^11^ ^]^

Photocatalyst	Method	H_2_ rate [mmol g^−1^.h^−1^]
Cu‐TiO_2_	Impregnation	17.3
	Photo deposition	24
	Hydrothermal	7
Pt/TiO_2_	Impregnation	58
	Photo deposition	36
	Hydrothermal	53

From Table 6 it can be seen that for Cu, the photo deposition and for Ni, the impregnation methods resulted in the maximum H_2_ production rates. The hydrogen production rate is influenced by both the loading method and the type of co‐catalyst used.^[^ ^11^ ^]^ Various techniques used to synthesize and modify TiO_2_ have been reported in the literature; the advantages and disadvantages of each technique are outlined in **Table** **7**.

**Table 7:** Advantages and disadvantages of different synthesis methods.

Synthesis method	Phases		Advantages	Disadvantages	References
	Anatase	Rutile			
Precipitation	✓	✓	Low temperature Simple process Large scale production	Difficult to control the particle size Easy agglomeration of the particles Low purity and large and uneven particle size.	[26, 30]
Solvothermal/ Hydrothermal	✓	✓	Easy control of surface and particle properties (size and shape). High purity, Homogeneity, High crystallinity Good dispersion Easy modification of the size of the doped photocatalyst.	Extended synthesis rime Requires expensive equipment Difficulties in mass production	[97, 100]
Sol‐gel	✓	✓	Doping flexibility Uniform chemical composition Controllable microstructure and homogeneity	Calcination stage leads to reduced surface area Long Process time Formation of agglomerates	[26, 30, 97, 100]
Micro‐emulsion	Amorphous		Simple equipment Controllable particles	Uncontrolled aggregation and flocculation. Difficulties in mass production	[30]
Chemical vapor deposition	✓	✓	High surface area particles Easy to scale up	Capital and energy‐intensive	[30]
Impregnation			High homogeneity Low operation cost	Agglomeration of particles	[97]

## 6 Photoreactors

**Figure 8:**
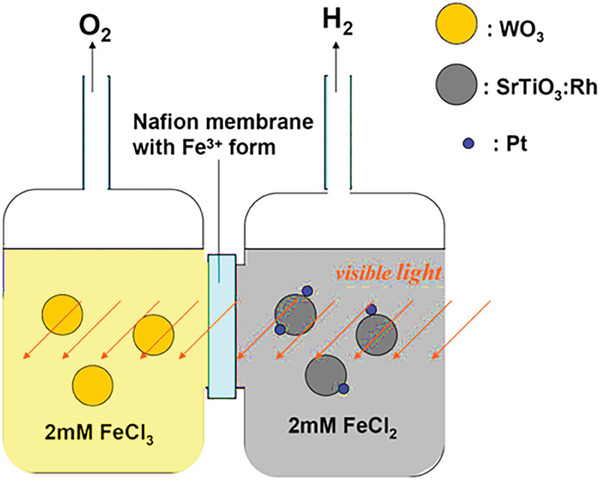
Schematic diagram of a twin reactor system.^[^ Reproduced with permission.^[^ ^99^ ^]^ Copyright 2010, Elsevier.

The type of reactor is a key factor that affects the process of photocatalytic hydrogen production.^[^ ^88^ ^]^ Photoreactor refers to the vessel in which a photocatalyst reacts with the reactant under light irradiation to produce products.^[^ ^18^ ^]^ The efficiency of the photocatalyst is influenced by the design and setup of the photocatalyst and light distribution within the reactor.^[^ ^17^ ^]^ Various types of photoreactors have been developed based on the factors such as the phase of the photocatalyst, the nature of reaction solution and operational requirements.^[^ ^98^ ^]^ Photocatalytic water splitting occurs in two fundamental reactor configurations based on the form of the photocatalysts: 1) Powder form suspended in liquid and 2) Photocatalysts immobilized onto continuous inert carriers.^[^ ^17^ ^]^ For optimal photocatalytic activity, an ideal photoreactor should ensure a consistent distribution of light throughout the photoreactor.^[^ ^18^ ^]^ Different positions of the light source that are reported are discussed in section 4.4. The batch reactor is the most extensively studied photo reactor utilizing a powdered photocatalyst.^[^ ^88^ ^]^ The summary of different types of reactors is given in **Table** **8**. The schematic diagram of an innovative twin‐reactor system facilitating the separate production of hydrogen and oxygen is presented in **Figure** **8**.

**Table 8:** Different types of photoreactors.^[^ ^17^, ^88^, ^98^, ^99^, ^101^, ^102^ ^]^

Reactor	Description	Advantages	Disadvantages
Batch reactor	Batch configuration	Possible to use an external light source Excellent irradiation distribution Effective mass transport Provides large surface area for effective photocatalysis	Challenges in separating the powdered catalyst from the reaction mixture Undesired back reaction
Slurry reactor	It comprises catalyst in particulate form. Employed when reactants are present in both gas and liquid phases.	Utilization of uniform external surface illumination throughout the reaction Can operate in either fixed bed mode or continuous flow pattern	Separating of catalyst particles from a mixture can be challenging Continuous stirring causes additional cost
Fluidized reactor	It is a fusion of a stirred tank and packed bed continuous flow reactors.	Significant photocatalytic efficacy Enhanced heat and mass transfer efficiency	Difficult Separation Abrasion and attrition of the particles may cause reactor erosion
Optical fiber reactor	Optical Fibers are utilized to evenly distribute light within a photoreactor by applying a coating of a photocatalyst onto the Fibers	Increased surface area High efficiency in light utilization Effective catalyst processing capabilities Economical operation	Catalyst deactivation at elevated temperatures Complexity in uniformly coating Fibers
Monolith reactor	It uses monolith support comprising homogenous blocks with parallel channels, which can be extruded into various shapes and sizes.	Increased surface‐to‐volume ratio Minimal pressure drop Enhanced flow rate	Reduced light efficiency Limited catalyst adhesion to the wall
Fixed bed reactor	It comprises solid catalyst particles loaded and packed into the bed.	Provides large surface area Accelerated reaction time High conversion efficiency per catalyst mass Economical operation	Limited exposure of the catalyst to light Low conversion and yield rate
Twin reactor system and H‐Type		Hydrogen and oxygen can be produced separately Reduced risk of backward reaction reduced cost of hydrogen separation before usage Reduced risk of explosion hence it is safe for commercial operation and scalability.	Increased cost

## 7 Quantum Yield

The effectiveness of photocatalytic hydrogen production through water splitting can also be evaluated by measuring the quantum yield which relies on the comprehensive quantum efficiency of the photocatalyst system; this allows quantifiable comparison between different photocatalysts.^[^ ^103^ ^]^ The quantum yield is a measure of energy efficiency used to assess the effectiveness of photon utilization in photochemical processes.^[^ ^104^ ^]^ For photocatalytic water splitting, quantum yield can be defined as the ratio of the hydrogen radical formation to the rate of absorbed photons (Equation (5)).^[^ ^105^ ^]^

Quantum yield can be calculated as follows.^[^ ^105^ ^]^

(5) $$QY_{H^{\cdot }}=\left[\frac{moles\;of\frac{\;H^{\cdot }}{s}}{moles\;of\;photons\;absorbed\;by\;the\frac{photocatalyst}{s}}\right]100$$

The apparent quantum yield (AQY) or Overall quantum yield of the H_2_ evolution reaction can be calculated using Equation (6).^[^ ^60^, ^103^ ^]^

(6) $$\begin{array}{ccc} AQY & = & \frac{Number\;of\;reacted\;electrons}{Number\;of\;incident\;photons\;}\;\;\times 100\%\\ & = & \frac{2\times Number\;of\;H_{2}\;molecules\;evolved\;per\;hour}{Number\;of\;incident\;photons\;per\;hour\;}\;\times 100\% \end{array}$$

Acar et al. performed a comparative assessment of the quantum yield and rate of hydrogen production among different photocatalysts.^[^ ^103^ ^]^ The results indicated that SrTiO_3_:Ni/La/Ta exhibited the highest quantum yield whereas TiO_2_‐ZnO resulted in the lowest quantum yield. Furthermore, in their comparison of H_2_ rates among different photocatalysts it was found that Au/TiO_2_ resulted in the highest H_2_ rate.^[^ ^103^ ^]^ Rusinque studied the effect of catalyst concentration and different metal loading on Quantum yields, and it was found that these factors significantly influence the quantum yield. From Table 3 it is evident that the highest AQY(43.8%) was achieved with WSe_2_‐TiO_2_ photocatalyst, however, only a limited number of studies reported the quantum yield.^[^ ^106^ ^]^

## 8 Challenges and Future Perspectives of Photocatalytic Water Splitting

Villa et al. reported the benefits and drawbacks of photocatalytic water splitting. The benefits include environmental friendliness, simple setup, and sustainability.^[^ ^29^ ^]^ However, the challenges of photocatalytic water splitting include 1) Product separation, 2) Low quantum efficiencies, and 3)Reproducibility and Scalability. During photocatalytic water splitting reactions hydrogen and oxygen are produced in the same reactor, this causes problems such as fast recombination rates and decreased photocatalytic efficiency. However, Z‐scheme photocatalyst (semiconductors coupling) which enables the separation of products has been researched and developed.^[^ ^29^ ^]^ Another potential solution to this challenge includes using novel photoreactor systems such as H‐type and twin reactor systems which allow the separation of the products.^[^ ^3^ ^]^ However, this results in increased costs and affects the economic viability of the process.^[^ ^29^ ^]^

One of the major drawbacks of PWS is low quantum efficiency. The cause of low efficiency is the reduced overall photocatalytic performance resulting from fast recombination rates. The photocatalytic performance is affected by many factors as discussed in Section 2.2.3. The approaches to reduce the recombination rates which results in enhanced hydrogen production rates include using co‐catalysts and catalysts with smaller particle size.^[^ ^3^ ^]^ Eidsvåg et al. reported that the size of the nanomaterials and co‐catalysts can modify the photocatalytic activity by reducing the recombination rates.^[^ ^6^ ^]^ Jagadeesh Babu et al. reported that the 1D nanostructures have fast transfer and efficient charge separation and 2D nanostructures have also shown improved photocatalytic activity and hydrogen production.^[^ ^75^ ^]^ Advancements in techniques have facilitated the deposition of ultra‐thin films, synthesis of nanoparticles of various sizes, and forms such as nanowires, nanorods, nanobelts and nanosheets.^[^ ^6^ ^]^

Another challenge in PWS is ensuring consistency in hydrogen production rate reported by various researchers, as the rate are influenced by the specific details of the experimental setup.^[^ ^14^ ^]^ Eidsvåg et al. reported that the suggestion and solution for a standard experimental setup are needed and one commonly proposed solution by various researchers is to report apparent quantum yield (AQY) rather than hydrogen generation rate because AQY is independent of the experimental details.^[^ ^6^ ^]^ As photocatalytic materials are used in powder form with small particle sizes, their separation from large volumes is challenging. The solution to this problem involves using immobilized photocatalyst systems.^[^ ^29^ ^]^ However, a major challenge of absence of scalable PWS systems for hydrogen production still remains. Notably, the large‐scale facilities that exist have extremely low solar to hydrogen efficiency of 1.5%, this indicates the necessity for further research advancements before photocatalytic hydrogen production through water splitting can compete with other methods of hydrogen production.^[^ ^3^ ^]^ Considering the challenges, it can be inferred that additional research and development efforts focusing on the potential photocatalysts and photoreactors capable of achieving the highest solar to hydrogen efficiency are necessary to advance photocatalytic water splitting toward industrial applications.

## Conflict of Interest

The authors declare no conflict of interest.
